# A new cirrhotic animal protocol combining carbon tetrachloride with methotrexate to address limitations of the currently used chemical-induced models

**DOI:** 10.3389/fphar.2023.1201583

**Published:** 2023-06-15

**Authors:** Rasha A. Mansouri, Adel M. Ahmed, Huda F. Alshaibi, Maha M. Al-Bazi, Abeer A. Banjabi, Hadeil Muhanna Alsufiani, Akram Ahmed Aloqbi, Esam M. Aboubakr

**Affiliations:** ^1^ Prince Sattam Bin Abdulaziz University, Al-Kharj, Saudi Arabia; ^2^ Biochemistry Department, Faculty of Sciences, King Abdulaziz University, Jeddah, Saudi Arabia; ^3^ Department of Pharmaceutical Analytical Chemistry, Faculty of Pharmacy, South Valley University, Qena, Egypt; ^4^ Embryonic Stem Cell Unit, King Fahd Medical Research Center, King Abdulaziz University, Jeddah, Saudi Arabia; ^5^ Experimental Biochemistry Unit, King Fahd Medical Research Center, King Abdulaziz University, Jeddah, Saudi Arabia; ^6^ Department of Biology, Faculty of Science, University of Jeddah, Jeddah, Saudi Arabia; ^7^ Department of Pharmacology and Toxicology, Faculty of Pharmacy, South Valley University, Qena, Egypt

**Keywords:** liver cirrhosis, carbon tetrachloride, methotrexate, cirrhotic animal model, micellar liquid chromatography

## Abstract

**Background:** Chemically induced cirrhotic animal models are commonly used. However, they have limitations such as high mortalities and low yield of cirrhotic animals that limit their uses.

**Aims:** To overcome limitations of the chemically induced cirrhotic animal model via combined administration of methotrexate (MTX) with CCl_4_ and decrease their commonly used doses depending on the proposed synergetic cirrhotic effect.

**Methods:** Rats were divided into six groups: normal (4 weeks), normal (8 weeks), MTX, CCl_4_ (4 weeks), CCl_4_ (8 weeks), and MTX + CCl_4_ (4 weeks) groups. Animals’ hepatic morphology and histopathological characterization were explored. Hepatic Bcl2 and NF-κB-p65 tissue contents were determined using the immunostaining technique, and hepatic tissue damage, oxidative status, and inflammatory status biochemical parameters were determined.

**Results:** CCl_4_ + MTX combined administration produced prominent cirrhotic liver changes, further confirmed by a substantial increase in oxidative stress and inflammatory parameters, whereas mortalities were significantly lower than in other treated groups.

**Conclusion:** The present study introduced a new model that can significantly improve the major limitations of chemically induced cirrhotic animal models with new pathological features that mimic human cirrhosis. Compared to other chemically induced methods, the present model can save time, cost, and animal suffering.

## 1 Introduction

Liver cirrhosis is a serious and life-threatening disease that is characterized by irreversible scarring of the liver (Ye et al., 2022). It is the final stage of liver fibrosis, which is the buildup of scar tissue in the liver (Koyama and Brenner, 2017; Aboubakr et al., 2022). Cirrhosis can be caused by a variety of factors, including viral infections, autoimmune disorders, alcohol abuse, and certain medications (Ye et al., 2022).

The development of cirrhosis is a complex process that involves a number of different factors. One of the key factors is the activation of stellate cells, which are a type of cell in the liver. When stellate cells are activated, they produce collagen, which is a major component of scar tissue (Zhou et al., 2014). Other factors that contribute to the development of cirrhosis include inflammation, oxidative stress, and apoptosis (cell death) (Nickovic et al., 2018; Tang et al., 2021; Pomacu et al., 2021).

Cirrhosis can lead to a number of serious complications, including liver failure, portal hypertension, and hepatocellular carcinoma (HCC). Liver failure occurs when the liver is no longer able to function properly. Portal hypertension is a condition in which pressure in the portal vein (a major vein that carries blood from the digestive tract to the liver) is increased. HCC is a type of liver cancer that is often associated with cirrhosis (Nusrat et al., 2014).

There is no cure for cirrhosis, but there are treatments that can help to slow the progression of the disease and prevent complications. Treatment may include medications, lifestyle changes, and liver transplantation (Nusrat et al., 2014).

Understanding the underlying mechanisms of liver cirrhosis is essential for developing new drugs to prevent and treat the disease. Animal models have been developed to mimic cirrhosis; however, to date, all existing models have low to moderate reproducibility, and none can fully replicate the human disease (Tropskaya et al., 2020; Chen et al., 2021).

Liver cirrhosis can be induced in rats in a number of ways. The most common methods involve the administration of chemical substances that cause cirrhotic changes (Natarajan et al., 2006; Bae et al., 2022) (Dong et al., 2016). These substances include carbon tetrachloride (CCl_4_) and thioacetamide (TAA) (Tropskaya et al., 2020). CCl_4_ and TAA are both hepatotoxic, meaning they damage the liver. When they are administered to rats, they can cause the liver to become scarred and dysfunctional. This protocol is effective in causing cirrhosis, but it can also be harmful to the rats.

In addition to the administration of chemical substances, liver cirrhosis can also be induced in rats by ligating the common bile duct. This procedure blocks the flow of bile from the liver, which can damage the liver and lead to cirrhosis. This protocol is less harmful to rats but less effective in causing cirrhosis (Ali et al., 2018).

Carbon tetrachloride (CCl_4_) can cause liver damage when it is administered to rats. This damage is caused by reactive metabolites that are generated by cytochrome P-450 liver enzymes, principally CYP2E1 (Fortea et al., 2018). When CCl_4_ is administered repeatedly, it can cause hepato-centrilobular necrosis, inflammation, and activation of hepatic stellate cells. This can lead to the upregulation of the synthesis of the extracellular matrix, architectural alterations in hepatic tissues, and, eventually, liver cirrhosis (Scholten et al., 2015).

The experimental model of CCl_4_-induced cirrhosis has several limitations, including high mortality rates, a limited number of animals that develop cirrhosis, toxicity risks, and a long time for induction (Scholten et al., 2015). Researchers have tried to overcome these limitations by using cytochrome P-450 inducers (Chatamra and Proctor, 1981), individualizing CCl_4_ doses according to body weight (Proctor and Chatamra, 1982; Regimbeau et al., 2008), and using different timetables for CCl_4_ administration (Runyon et al., 1991; Desmyter et al., 2007; Regimbeau et al., 2008). However, these problems still persist.

Methotrexate (MTX) has been used in animal models to induce liver damage for many years (Garcia et al., 2019). High-dose MTX can cause hepatic steatosis and fibrosis (Garcia et al., 2019). The exact mechanism of MTX-induced hepatotoxicity is not fully understood, but it may involve the generation of reactive oxygen species (ROS) and lipid peroxidation. ROS can induce the release of proinflammatory cytokines, which can damage hepatocytes and lead to apoptosis (Garcia et al., 2019).

MTX can also inhibit the production of reduced nicotinamide adenine dinucleotide phosphate (NADPH), which is a molecule that is essential for the production of glutathione (GSH). GSH is a powerful antioxidant that helps to protect cells from damage (Shetty et al., 2017). When MTX inhibits the production of NADPH, it can lead to a decrease in GSH levels, which can make cells more susceptible to damage. This damage can lead to the death of hepatocytes, which are the cells that make up the liver (Sahindokuyucu-Kocasari et al., 2021).

The present study was conducted to introduce a new animal model of liver cirrhosis that overcomes some limitations of the CCl_4_-induced cirrhosis model. The new model uses the combined administration of MTX and CCl_4_, which is based on the proposed synergism of these two drugs. The new model has a lower mortality rate and a shorter induction time than the CCl_4_-induced model, and it produces more cirrhotic animals. The study also developed a new and validated HPLC method for detecting and quantifying MTX concentration in both *in vitro* and in hepatic tissue.

## 2 Materials and methods

### 2.1 Chemicals and reagents

MTX, sodium acetate, disodium hydrogen phosphate, thiobarbituric acid, and sodium dodecyl sulfate (SDS) were purchased from Sigma-Aldrich (Saint Louis, MO, United States). HPLC-grade methanol, CCl_4_, and acetonitrile were purchased from Merck, Darmstadt, Germany.

### 2.2 Animals

This research was designed and performed according to ethical standards approved by the ethical committee at the Faculty of Pharmacy, South Valley University (approval # P.S.V.U212).

Animals were purchased from Helwan Animal Breeding House, Cairo, Egypt. A total of 84 adult male Sprague–Dawley rats weighing 125–140 g were used. The animals were fed standard rat chow and water *ad libitum* and kept under standardized conditions with 12 h light–12 h dark cycle at 26°C ± 2°C temperature. The animals were handled according to international and national ethical guidelines. The rats were randomly divided into six groups, 14 animals each, as follows:

Group I: animals served as normal control, whereas animals received corn oil (1 mL/kg, i.p., twice a week) for 4 consecutive weeks and normal saline (0.5  mL, i.p.) at a single dose.

Group II: animals served as normal control, whereas animals received corn oil (1 mL/kg, i.p., twice a week) for 8 consecutive weeks and normal saline (0.5   mL, i.p.) at a single dose.

Group III: animals received 20 mg/kg i.p. of MTX as a single dose (Yucel et al., 2017).

Group IV: animals received CCl_4_ as 50% solution in corn oil (1 mL/kg, i.p., twice a week) for 4 consecutive weeks (Aboubakr et al., 2022).

Group V: animals received CCl_4_ as 50% solution in corn oil (1 mL/kg, i.p., twice a week) for 8 consecutive weeks (Aboubakr et al., 2022).

Group VI: animals received 10 mg/kg i.p. of MTX as a single dose plus CCl_4_ as 50% solution in corn oil (0.5 mL/kg, twice a week i.p.) for 4 consecutive weeks.

During the experiment, the number of deaths between different animal groups was determined. At the end of the experimental periods, the animals were weighed, deeply anesthetized with thiopental sodium, and then blood was collected from the inferior vena cava into clean tubes. The animals were then sacrificed by cervical dislocation. The collected blood samples were centrifuged at 3,000 rpm for 10 min. The liver was washed with ice-cold saline (0.9% w/v) solution, blotted dry, and then weighed. Portions of the liver were placed in ice-cold 0.1 M phosphate buffer (pH 7.4) and homogenized. After homogenization, the samples were centrifuged at 5,000 rpm for 10 min.

### 2.3 Chromatographic analysis

#### 2.3.1 Instruments and conditions

The analytical method was developed using the Agilent 1260 Infinity II^®^ HPLC System (Agilent Technologies, Germany) equipped with a Pursuit-3 C18 column (150 × 4.6  mm, 3 µm) maintained at 30°C. The output signals were observed and processed using ChemStation^®^ software.

The mobile phase consisted of sodium acetate buffer (50 mM, pH 3.5) containing SDS (2.67 mM) and acetonitrile (85:15, v/v). The chromatographic run was carried out in isocratic mode with a flow rate of 0.5 mL/min. The injection volume and the detection wavelength were 20 µL and 290 nm, respectively.

#### 2.3.2 Preparation of stock and working solutions

The stock solution of MTX was prepared in the mobile phase at 10.0 mg/mL and was stored at −20°C for further analysis. Working solutions, at 0.5 μg/mL, were freshly prepared by dilution of the stock solution with the mobile phase.

#### 2.3.3 Calibration of MTX in tissue homogenates

To an aliquot of 0.5 mL of liver homogenates (free from MTX), appropriate volumes of MTX working solution were added at concentrations of 2.5–78.0 μg/mL. The mixtures were vortex-mixed for 3 min and centrifuged at 6,000 rpm for 5 min at room temperature. Then, 20 µL aliquot of clear supernatant was injected into the HPLC system for analysis.

### 2.4 Immuno-histopathological examination

Rats’ hepatic tissue samples were fixed in 10% formalin at room temperature for 24 h. The samples were then dehydrated using graded ethanol and embedded in paraffin wax. The hepatic tissues were sectioned with a thickness of 5  μm and deparaffinized using xylene. The sections were stained using hematoxylin and eosin (H&E) and Masson trichrome stains. To quantify the fibrotic tissue, the images of the blue pixels were photographed. Eight different areas from each slide for each rat were selected to determine the values of the integral optical density and the total area. The expression intensity was estimated as the percentage of the integral optical density to the total area using the ImagePro Plus 6.0 program (Das et al., 2006). Pathological change scoring was assessed as described by Kleiner et al. (2005) with separate scores for steatosis (0–3), hepatocellular ballooning (0–2), and lobular inflammation (0–3).

To stain hepatic tissue for tumor necrosis factor-alpha (NF-κB-p65) and B-cell lymphoma 2 (Bcl-2), antibodies were obtained from Thermo Fisher Scientific Inc./Lab Vision (CA, United States) and used in a standard immune-histochemical procedure (El-Kashef and Serrya, 2019; Missaoui et al., 2019). Briefly, 4-μm-thick rat hepatic sections were deparaffinized and incubated in citrated buffer at pH 6.0 for 30 min at 100°C. After cooling, the sections were incubated for 12 h with a 1:200 dilution of each antibody in phosphate-buffered saline (PBS) solution, followed by repetitive rinsing using PBS. The bound antibodies were detected using avidin biotinylated peroxidase, followed by appropriate washing using PBS and counterstaining using Mayer’s hematoxylin.

Histopathological analysis was performed in the Pathology Department at the Faculty of Medicine, South Valley University. The tissue sections were visualized using an Olympus microscope with ×100 and ×400 magnifications. Quantification of NF-κB-p65 and Bcl-2 immune expression was performed using ImageJ^®^ software (National Institutes of Health, Bethesda, United States) by an expert pathologist (Aslan et al., 2018).

### 2.5 Hepatic tissue total protein content determination

The protein content of the hepatic tissue homogenates was determined using the Bradford assay (Kruger, 1994).

### 2.6 Liver enzyme determination

The activities of serum aspartate aminotransferase (AST) and alanine aminotransferase (ALT) were determined spectrophotometrically using commercial kits from Biodiagnostic Company, Egypt. The kits were used according to the manufacturer’s protocol (Huang et al., 2006).

### 2.7 Nitric oxide determination

Nitric oxide (NO) levels in hepatic tissue were determined using a commercial kit (Biodiagnostics, Cairo, Egypt). The kit uses a method that converts NO to nitrous acid, which then reacts with sulfanilamide and N-(1-naphthyl) ethylenediamine to form an azo dye. The absorbance of the dye is then measured spectrophotometrically at 540 nm (Bryan and Grisham, 2007).

### 2.8 Assessment of superoxide dismutase activity

Hepatic superoxide dismutase (SOD) activity was determined using a standard kit (Biodiagnostics, Cairo, Egypt) according to the manufacturer’s instructions. Serial dilutions of the standard SOD and samples were added to each well, followed by the radical detector and xanthine oxidase. The plate was shaken and incubated at room temperature for 30 min. The absorbance of the samples was recorded at 440–460 nm using an ELISA microplate reader (Okado-Matsumoto and Fridovich, 2001).

### 2.9 Assessment of catalase activity

Catalase (CAT) activity in hepatic tissue homogenates was determined using a commercial kit (Biodiagnostics, Cairo, Egypt). The kit uses a method that measures the rate of decomposition of hydrogen peroxide (H_2_O_2_) in the presence of CAT. The amount of H_2_O_2_ that is decomposed is proportional to the amount of CAT in the sample. The color of the reaction product is then measured at 620 nm (Noeman et al., 2011).

### 2.10 Reduced glutathione determination

The hepatic tissue content of GSH was determined using a standard kit (Biodiagnostics, Cairo, Egypt). The kit uses a method that measures the formation of 5-thio-2-nitrobenzoate (TNB). The amount of TNB that is formed is proportional to the amount of GSH in the sample. The absorbance of the TNB solution is then measured spectrophotometrically at 412 nm (Noeman et al., 2011).

### 2.11 Inflammatory cytokine determination

The hepatic content of the inflammatory cytokines, namely, interleukin 1 beta (IL-1β) (Gieling et al., 2009), tumor necrosis factor-alpha (TNF-α) (Lai et al., 2013), and interleukin-6 (IL-6) (Bode et al., 2012) were determined using hepatic tissue homogenates and corresponding rat-specific ELISA assay kits according to the manufacturer’s protocols (Abcam, United Kingdom).

### 2.12 Malondialdehyde determination

Malondialdehyde (MDA) levels in hepatic tissue homogenates were determined spectrophotometrically by measuring the pink color produced by the reaction between MDA and thiobarbituric acid at 532 nm (Knockaert et al., 2012).

### 2.13 Determination of hydroxyproline in the hepatic tissues

Hydroxyproline was determined using the method described by Shin et al. (2018). Briefly, hepatic tissue homogenates were hydrolyzed by adding 6N HCl in a tightly capped glass tube and incubating overnight at 100°C. The acid hydrolysates were filtered, air-dried, and dissolved in methanol. 50% isopropanol and chloramine-T solution were added to each sample, followed by incubation at 37°C for 10 min. Ehrlich’s solution was added, followed by incubation at 50°C for 90 min. The optical density was determined at 558 nm using a spectrophotometer. Concentrations were then determined by comparison with a standard curve constructed using a serial dilution of hydroxyproline solution.

### 2.14 Statistical analysis

All parameters are expressed as the mean ± SD. Obtained results were statistically analyzed using a one-way ANOVA test, followed by Tukey’s multiple comparison test. Statistical significance was determined at *p* < 0.05. Analysis was performed using the GraphPad Prism software package (GraphPad Software, San Diego, CA 92108, United States).

## 3 Results

### 3.1 HPLC analysis and validation

Micellar liquid chromatography (MLC) is a technique that has several advantages over other methods of chromatography. It is relatively inexpensive, versatile, and can be used to analyze a wide variety of compounds, including those with different polarities. Additionally, MLC is considered to be a “green” technique because it uses less organic solvent than other methods, making it less harmful to the environment (Patyra and Kwiatek, 2021).

In this study, MLC has been used to analyze MTX in liver tissue homogenates without the need for extensive sample preparation. This is because micelles can competitively bind proteins and release protein-bound drugs without sedimentation on the column. This allows for direct analysis of the sample without the need for toxic or flammable organic solvents. This reduces the cost and analysis time and improves the separation efficiency.

The retention and selectivity of MTX in high-performance liquid chromatography can be affected by various factors, such as the pH of the mobile phase, temperature of the column, percentage of acetonitrile in the mobile phase, and concentration of SDS. These factors should be considered when developing a method for the analysis of MTX as follows:

**pH**: the retention time of MTX increases as the pH of the mobile phase decreases. This is because MTX is less ionized at lower pH, which makes it more hydrophobic and less soluble in the mobile phase.

**Temperature**: the retention time of MTX decreases as the temperature of the column increases. This is because the rate of mass transfer increases as the temperature increases.

**Acetonitrile**: the retention time of MTX decreases as the percentage of acetonitrile in the mobile phase increases. This is because acetonitrile is a polar solvent that can interact with the polar functional groups of MTX.

**SDS**: the retention time of MTX increases as the concentration of SDS in the mobile phase increases. This is because SDS is a surfactant that can interact with the hydrophobic regions of MTX. These findings can be used to optimize the conditions for the analysis of MTX by MLC.

**FIGURE 1:**
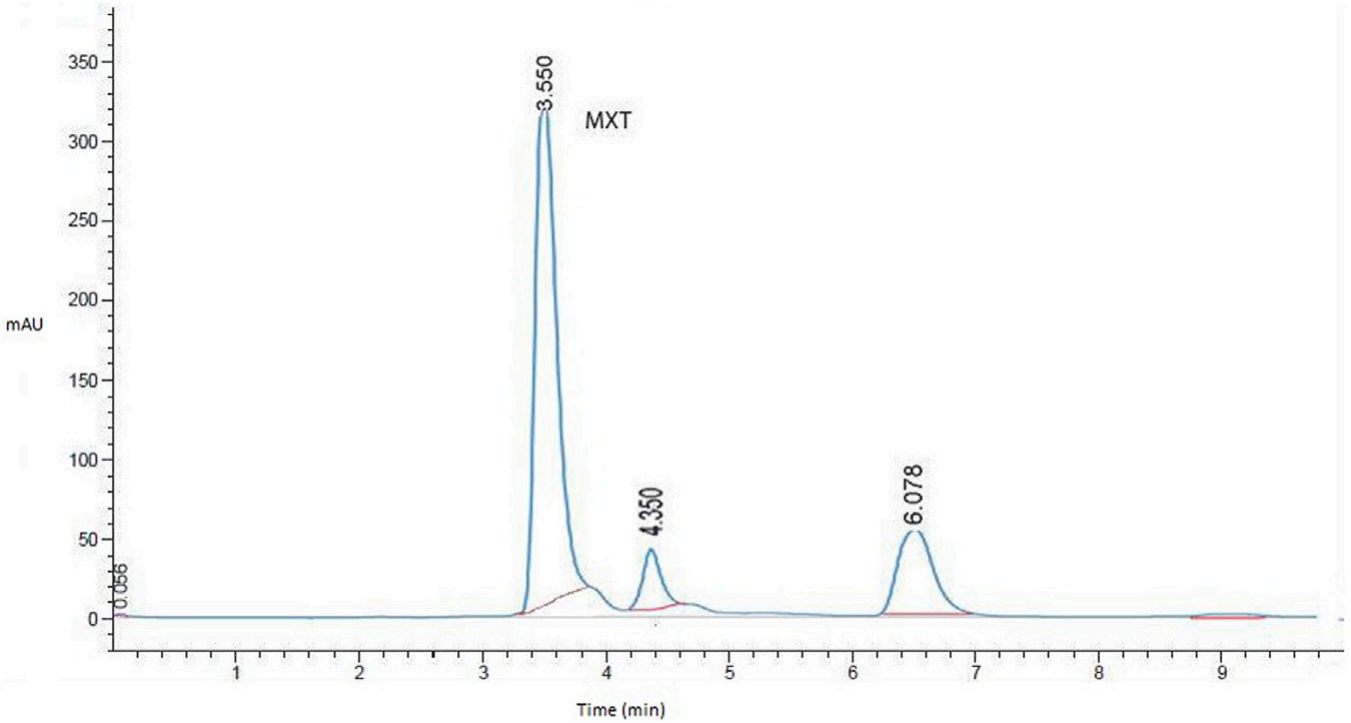
Representative HPLC chromatogram for MTX in liver homogenates (working standard mixture, 10.0 µgmL^−1^) taken under optimized conditions.

After several attempts to optimize the mobile phase, the optimum mobile phase for the analysis of MTX was found to be a mixture of sodium acetate buffer (50 mM, pH 3.5 containing 2.67 mM SDS) and acetonitrile (85:15). The column temperature was set at 30°C, the flow rate was 0.5 mL/min, and the detection wavelength was 290 nm. The retention time for MTX was 3.56 min (Figure 1).

The developed HPLC method was validated according to the International Council on Harmonization (ICH) guidelines (Bhavna et al., 2022). The calibration curve was linear over the concentration range of 2.5–78.0 μg/mL, with a correlation coefficient of more than 0.999. The limit of detection (LOD) and limit of quantitation (LOQ) were 0.75 and 2.28 μg/mL, respectively (Table 1). The inter-day (between days) and intra-day (within 1 day) precision values were less than 2%, and the accuracy of the method was in the range of 98.36%–102.03% (Table 2).

**TABLE 1:** Statistical parameters for individual calibration curves.

Parameter	Value
Λmax	290 nm
Linearity range (µgmL^−1^)	2.5–78.0
LOD (µgmL^−1^)	0.753
LOQ (µgmL^−1^)	2.28
*R* ^2^	0.9999
Regression equation	A٭ = bx٭٭ + a
Slope (x10^6^) ± SD	516.84 ± 0.022
Intercept (×10^6^) ± SD	180.11 ± 0.047

*A: absorbance; b: slope; x: concentration; a: intercept.

**TABLE 2:** Intra- and inter-day precision and accuracy of MTX evaluated by the developed micellar HPLC method.

Parameter	MTX concentration (µgmL^−1^)	% RSD^a^	% recovery ± SD^a^
Intra-day	3	1.503	99.63 ± 1.49
	30	0.641	99.45 ± 0.63
	70	0.719	100.53 ± 0.44
Inter-day	3	0.947	99.32 ± 0.94
	30	0.832	100.12 ± 0.85
	70	1.44	100.36 ± 1.45

^a^ RSD: relative standard deviation.

The system suitability parameters were also calculated and were found to be within acceptable limits. The tailing factor was 1.55, the asymmetry factor was 1.10, the theoretical plate number was greater than 2,000, and the height equivalent to a theoretical plate (HETP) was 0.00562 cm (Table 3). The well-shaped peaks in the chromatograms verified that the proposed method had satisfactory specificity (Patyra and Kwiatek, 2021).

**TABLE 3:** System suitability parameters for the determination of MTX by the micellar HPLC method.

HPLC parameter	MTX	Acceptable limits
Asymmetry factor	1.10	<1.5
Theoretical plates (m)	2,667	>200
Tailing factor	1.55	<2.0
HEPT^a^ (cm)	0.0056	

^a^ HEPT: height equivalent to a theoretical plate.

The developed HPLC method was used to determine the MTX concentrations in the hepatic tissue homogenates. The total amounts of MTX recovered were 598.95 ± 1.4 and 760 ± 0.96 µgmL^−1^ for group II and group IV, respectively.

### 3.2 Macroscopic examination of the hepatic organs

**FIGURE 2:**
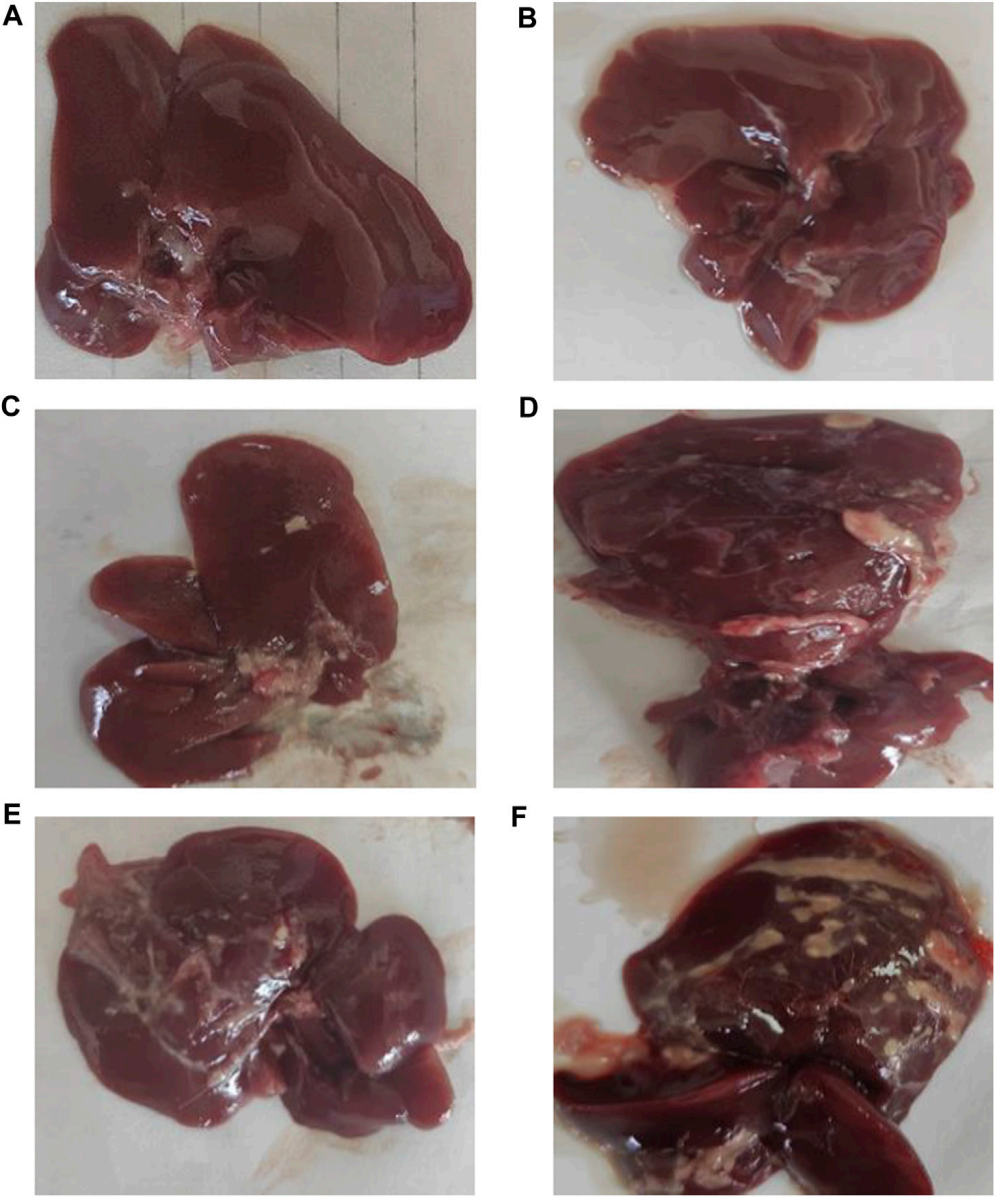
Macroscopic examination of the liver tissues from **(A)** normal rats treated for 4 weeks; **(B)** normal rats treated for 8 weeks; **(C)** methotrexate-treated rats; **(D)** carbon tetrachloride-treated rats for 4 weeks; **(E)** CCl4-treated rats for 8 weeks; and **(F)** CCl4 + MTX-treated rats for 4 weeks.

Figure 2 shows the typical hepatic architecture with normal gross morphology observed in the normal group at 4 weeks and 8 weeks. The CCl_4_ (4 weeks) animal group showed a pale and irregular surface, while the i.p. injection of CCl_4_ for 8 weeks caused an irregular hepatic surface with small nodules. Additionally, MTX i.p. administration showed a normal surface and gross morphology. On the other hand, MTX + CCl_4_ coadministration produced significant morphologic changes in rats’ livers, with a coarse pale irregular surface, shrunken cirrhotic changes, and multiple macro- and micronodules.

### 3.3 Effect on the liver index

**FIGURE 3:**
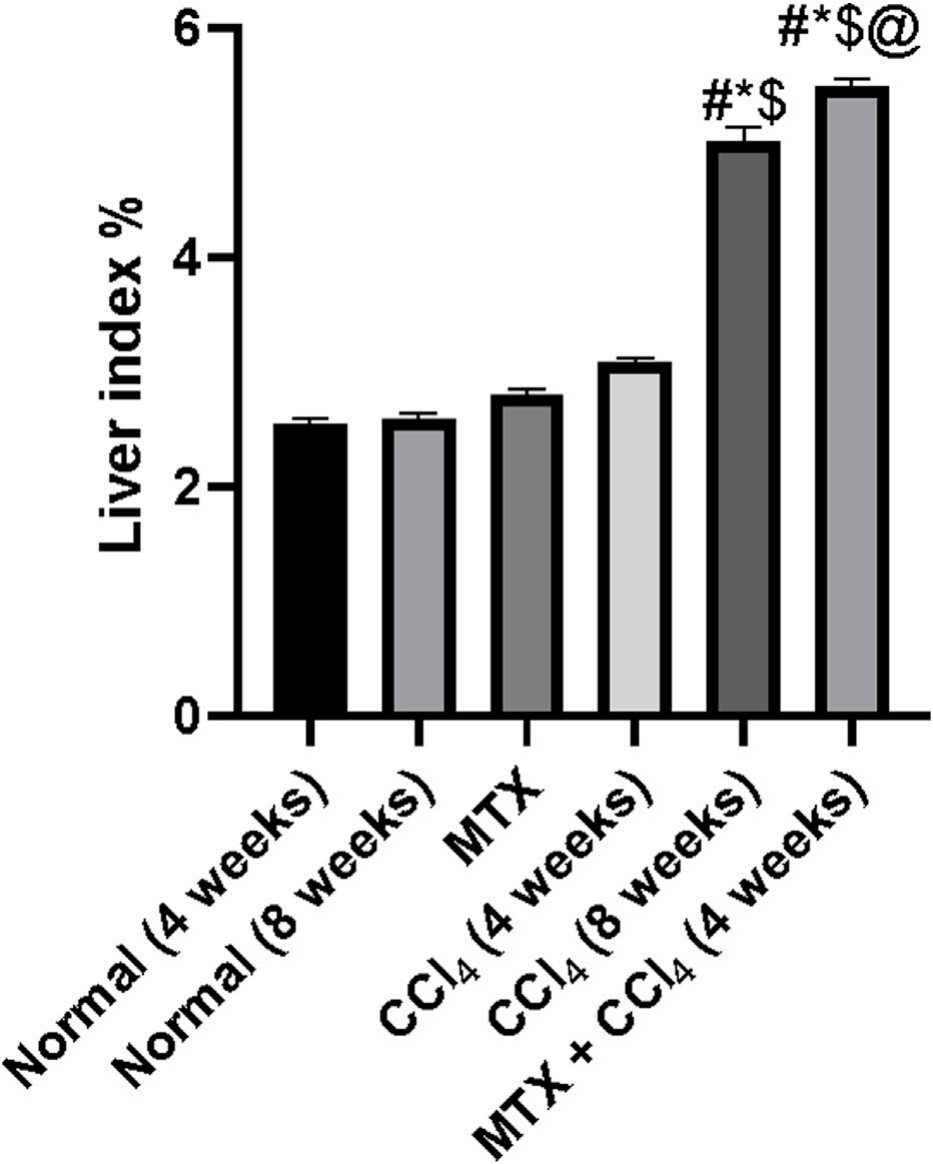
Effect of methotrexate, carbon tetrachloride, and their combined administration on the liver index. # = significantly different compared to the normal group, * = significantly different compared to the MTX group, $ = significantly different compared to the CCl_4_ (4 weeks) group, and @ = significantly different compared to the CCl_4_ (8 weeks) group. Results are presented as mean ± SEM (*n* = 14).

Rats treated with MTX and CCl_4_ for 4 weeks had a moderate increase in the liver index (liver wet weight/body weight × 100%) (Lin et al., 2014) compared to those from the control group (*p* < 0.05). On the other hand, rats treated with CCl_4_ for 8 weeks had a dramatic increase in the liver index, which was due to a decrease in body weight accompanied by an increase in liver weight. Moreover, the combined administration of MTX with CCl_4_ for 4 weeks further increased the liver index compared to that of the CCl_4_ (8 weeks) group, as shown in Figure 3.

### 3.4 Histopathological examination

#### 3.4.1 H&E staining

**FIGURE 4:**
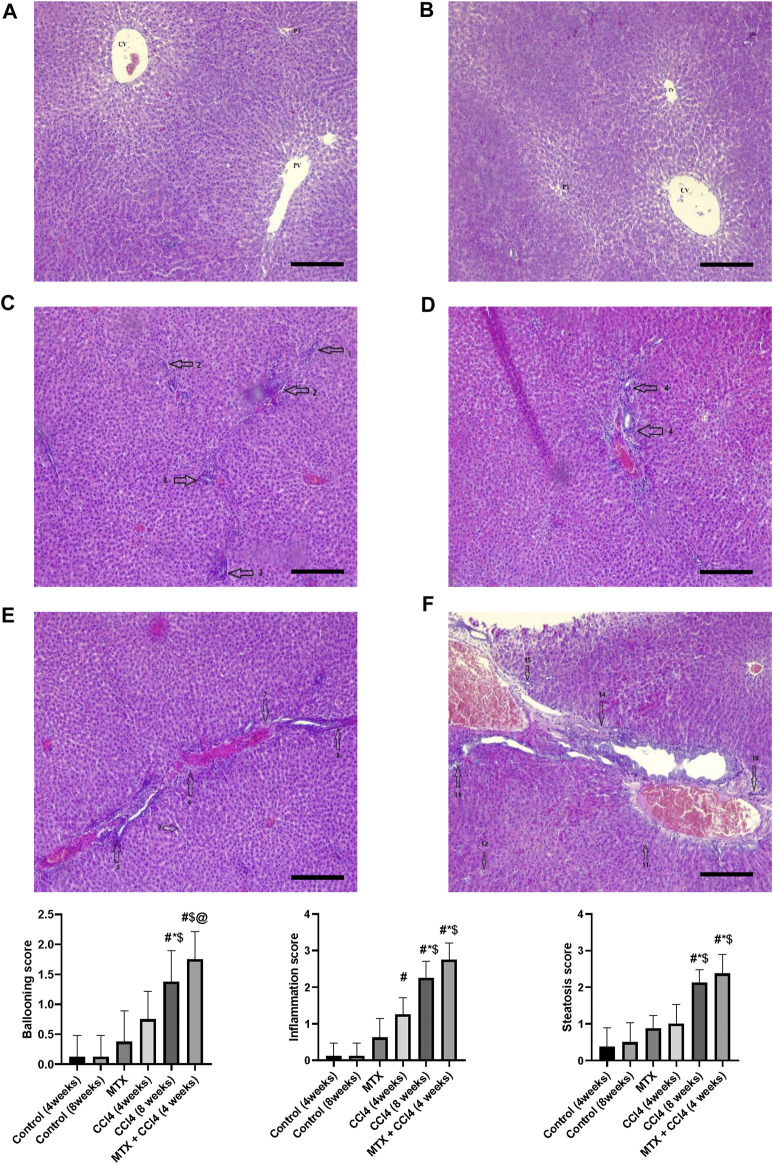
Histopathology of rats’ liver (H&E stain) from **(A)** normal rats treated for 4 weeks; **(B)** normal rats treated for 8 weeks; **(C)** methotrexate-treated rats; **(D)** carbon tetrachloride-treated rats for 4 weeks; **(E)** CCl4-treated rats for 8 weeks; and **(F)** CCl4 + MTX-treated rats for 4 weeks; the scale bar is 100 microns (CV = central vein, PV = portal vein, PT = portal tract, 1: moderate parenchymal cell changes, 2: mild dilatation of the sinusoidal space, 3: few inflammatory cell infiltrations, 4: inflammatory cell infiltration and mild congestion of central and portal veins, 5: sever portal inflammation, 6: centrilobular necrosis, 7: bundles of collagen, 8: intralobular degeneration, 9: advanced dilation of the sinusoidal space, 10: intense inflammatory cell infiltration, 11: hepatocyte steatosis, 12: dysplastic hepatocytes, 13: focal necrosis, 14: severe bridging fibrosis, and 15: hepatocyte ballooning).

The normal groups (4 and 8 weeks) showed normal hepatic tissue architecture, with normal hepatocytes radiating from the central vein (Figures 4A, B). The MTX-treated group showed moderate parenchymal cell changes, with mild dilatation of the sinusoidal space and a few inflammatory cell infiltrations (Figure 4C). The i.p. administration of CCl_4_ for 4 weeks produced marked congestion in the central vein, with moderate inflammatory cell infiltration and mild congestion of central and portal veins (Figure 4D). The i.p. injection of CCl_4_ for 8 weeks caused significant hepatic fibrosis, mild steatosis, marked inflammatory cell infiltration, and mild liver cirrhotic changes (Figure 4E). However, the combined administration of CCl_4_ with MTX caused an intense inflammatory cell infiltration, steatosis and degradation of hepatocytes, dysplastic hepatocytes, nodules with intense congestion of central and portal veins, focal necrosis, prominent hepatic tissue cirrhotic changes, and diffused Kupffer cell proliferation (Figure 4F).

#### 3.4.2 Masson’s trichrome stain

**FIGURE 5:**
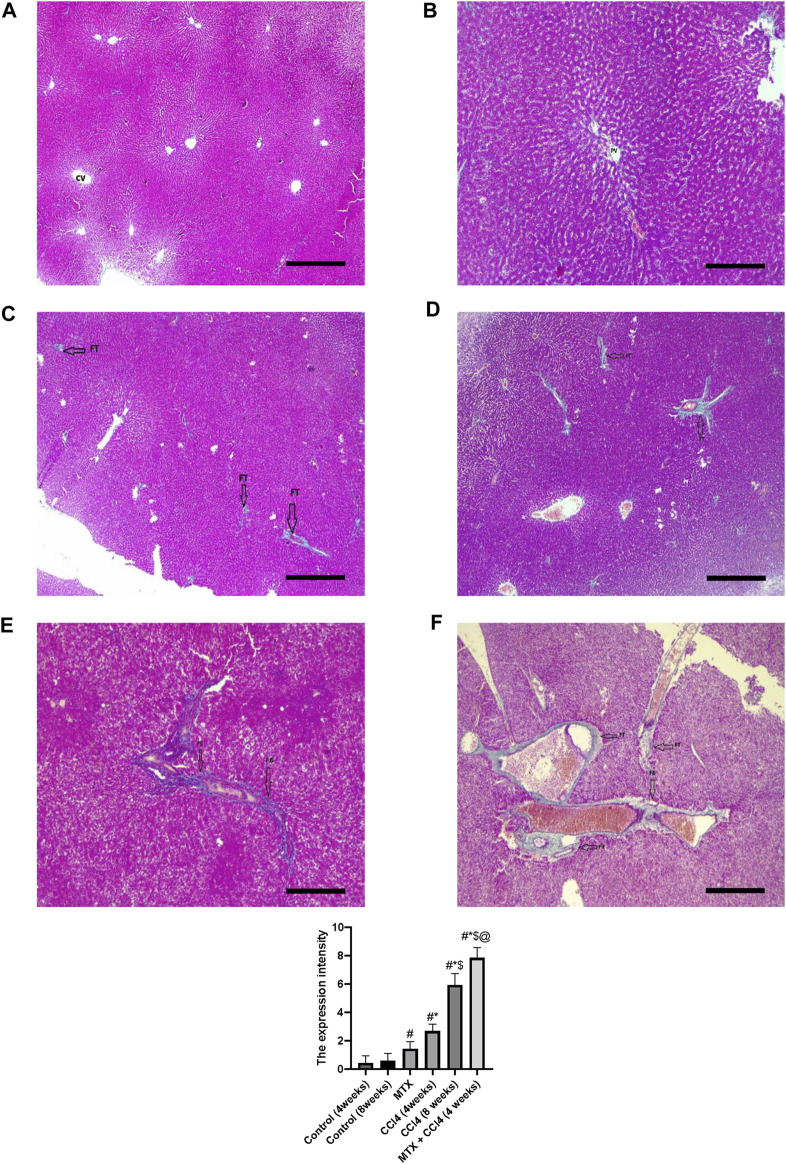
Histopathology of rats’ liver (Masson trichrome stain) from **(A)** normal rats treated for 4 weeks; **(B)** normal rats treated for 8 weeks; **(C)** methotrexate-treated rats; **(D)** carbon tetrachloride-treated rats for 4 weeks; **(E)** CCl4-treated rats for 8 weeks; and **(F)** CCl4 + MTX-treated rats for 4 weeks; the scale bar is 100 microns, (CV = central vein, PV = portal vein, FB = fibrous band, and FT = fibrous tissue).

Using Masson’s trichrome staining, the hepatic tissue samples of normal groups (4 and 8 weeks) showed weak staining of fibrous tissue at the central vein of hepatic lobules with normal hepatic tissue architecture (Figures 5A, B). In the MTX group, the collagen fibers in the liver were slightly increased compared to the normal group, with rare fibrous septa of the hepatic lobules (Figure 5C). The CCl_4_ (4 weeks) group showed a significant upregulation of fibrous tissue with fibrous septa of the hepatic lobules (Figure 5D). The CCl_4_ (8 weeks) group showed a significant upregulation of fibrous tissue deposition around the central vein and portal areas, which interacted with neighboring septa with intense blue coloration (where collagen is blue stained) (Figure 5E). The MTX + CCl_4_-treated group showed an intense fibrous tissue distribution within the hepatic tissue with pseudo broad lobe separation that connected the portal tract to the central vein and deposition of extensive thick collagen fibers in the hepatic parenchyma, which represents a distinct feature of cirrhotic liver changes (Figure 5F).

### 3.5 Effect on Bcl-2 hepatic tissue content

**FIGURE 6:**
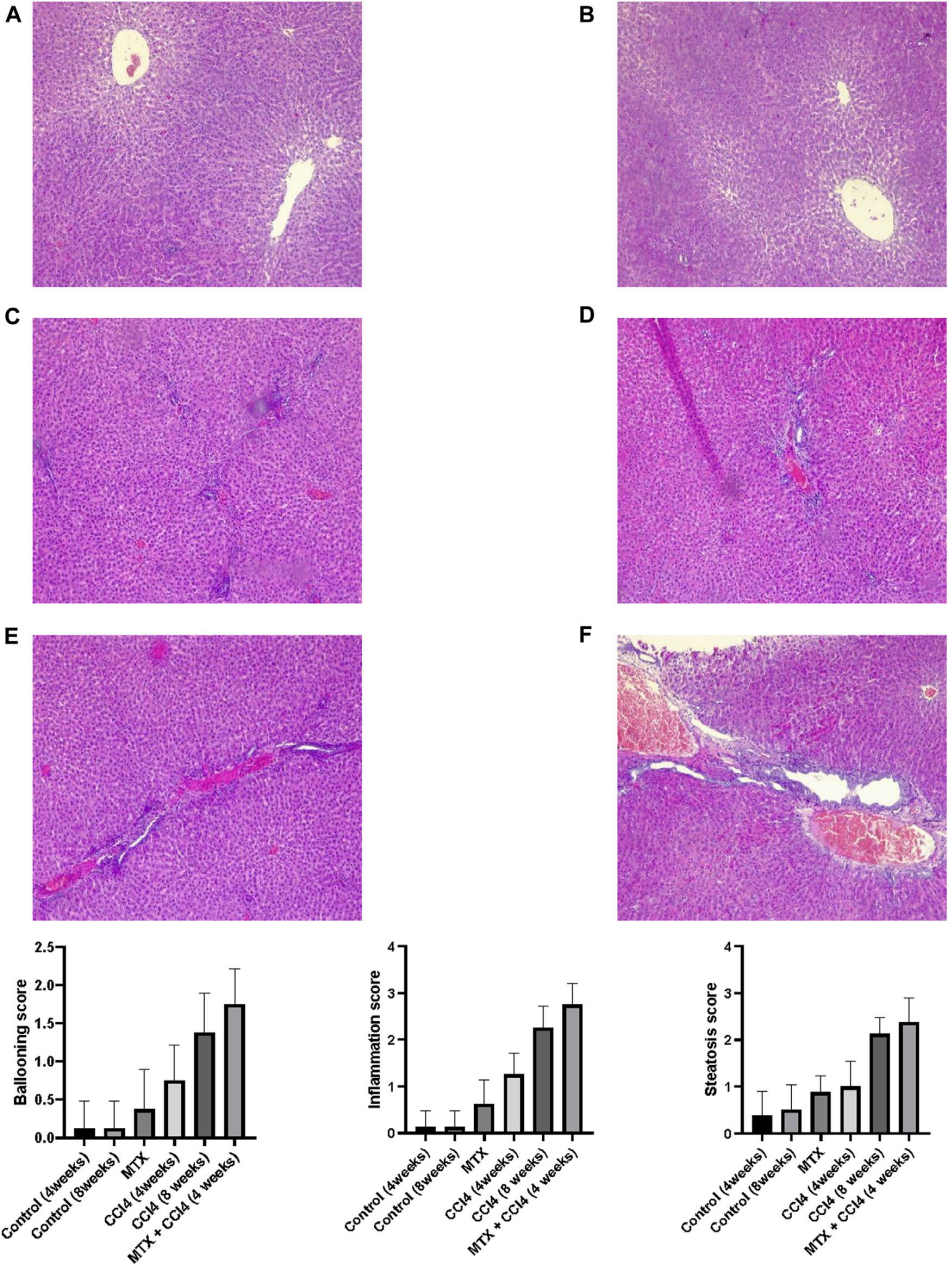
Immunohistochemistry results of Bcl-2 on hepatic tissues from **(A)** normal rats treated for 4 weeks; **(B)** normal rats treated for 8 weeks; **(C)** methotrexate-treated rats; **(D)** carbon tetrachloride-treated rats for 4 weeks; **(E)** CCl4-treated rats for 8 weeks; and **(F)** CCl4 + MTX-treated rats for 4 weeks.

The hepatic tissue distribution of Bcl-2 protein was determined using the immunostaining technique. The results showed that the hepatic tissue of normal groups showed many positive cells and a strong positive reaction (Figures 6A, B), which was nearly absent in the MTX + CCl_4_ (4 weeks) group (Figure 6F) and the CCl_4_ (8 weeks) group (Figure 6E). On the other hand, the MTX and CCl_4_ (4 weeks) groups showed a moderate distribution of Bcl-2 proteins with marked positive staining, which was more noticeable in the MTX group compared to the CCl_4_ group (Figures 6C, D).

### 3.6 Effect on NF-κB-p65 hepatic tissue content

**FIGURE 7:**
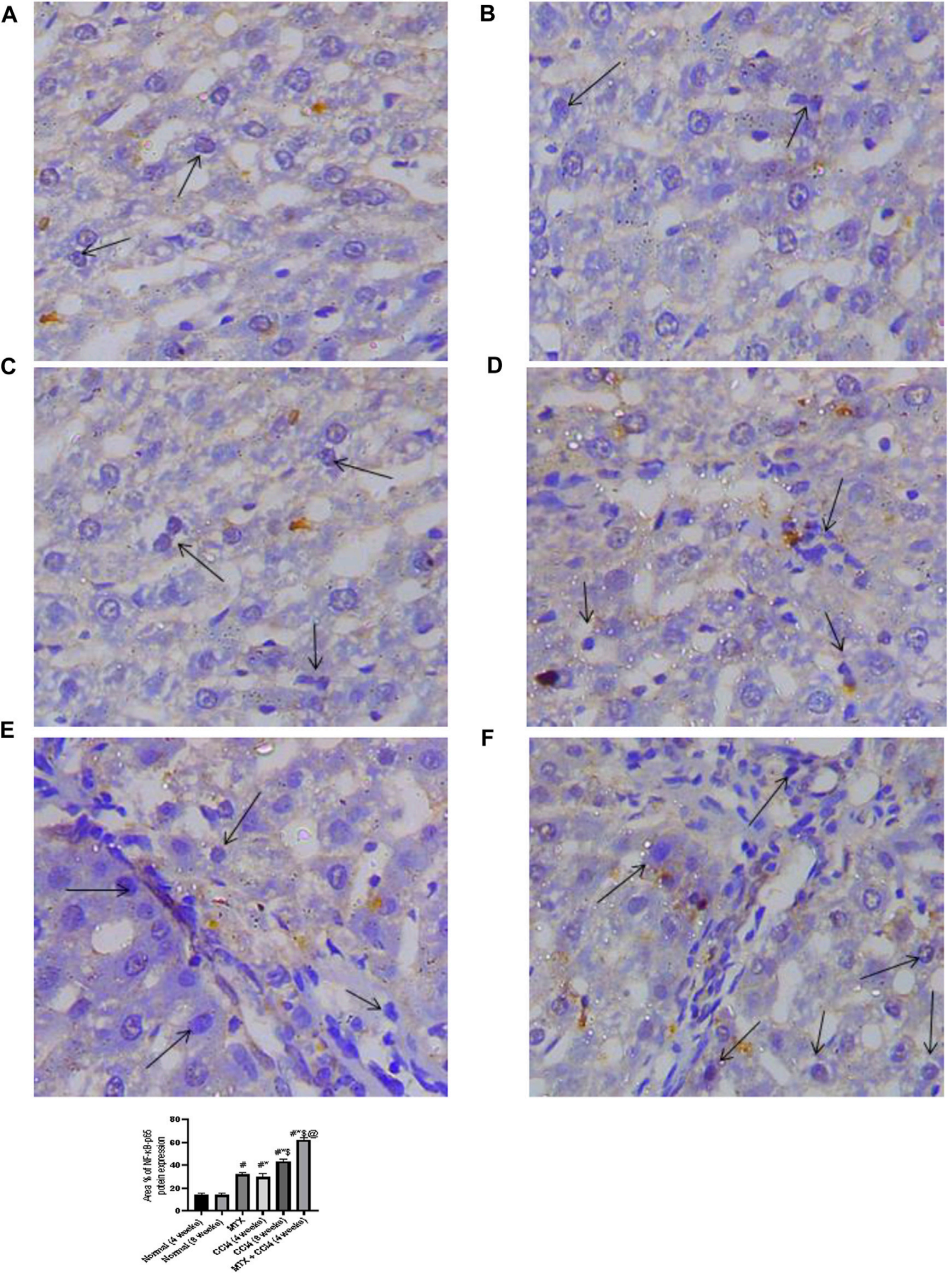
Immunohistochemistry results of NF-Kβ on hepatic tissues from **(A)** normal rats treated for 4 weeks; **(B)** normal rats treated for 8 weeks; **(C)** methotrexate-treated rats; **(D)** carbon tetrachloride-treated rats for 4 weeks; **(E)** CCl4-treated rats for 8 weeks; and **(F)** CCl4 + MTX-treated rats for 4 weeks.

NF-κB-p65 protein distribution was detected using the immunostaining technique. The results showed that the NF-κB-p65 protein was nearly absent in both normal groups (4 and 8 weeks) and moderately distributed in the MTX group. The i.p. administration of CCl_4_ for 4 weeks significantly upregulated the inflammatory mediator NF-κB-p65, with many positively detected hepatocytes. However, the combined administration of MTX with CCl_4_ dramatically increased the number of NF-κB-p65-positive hepatocytes with an intense reaction, which was more noticeable than in the CCl_4_ (8 weeks) group, as shown in Figure 7.

### 3.7 Effect on GSH hepatic content

**FIGURE 8:**
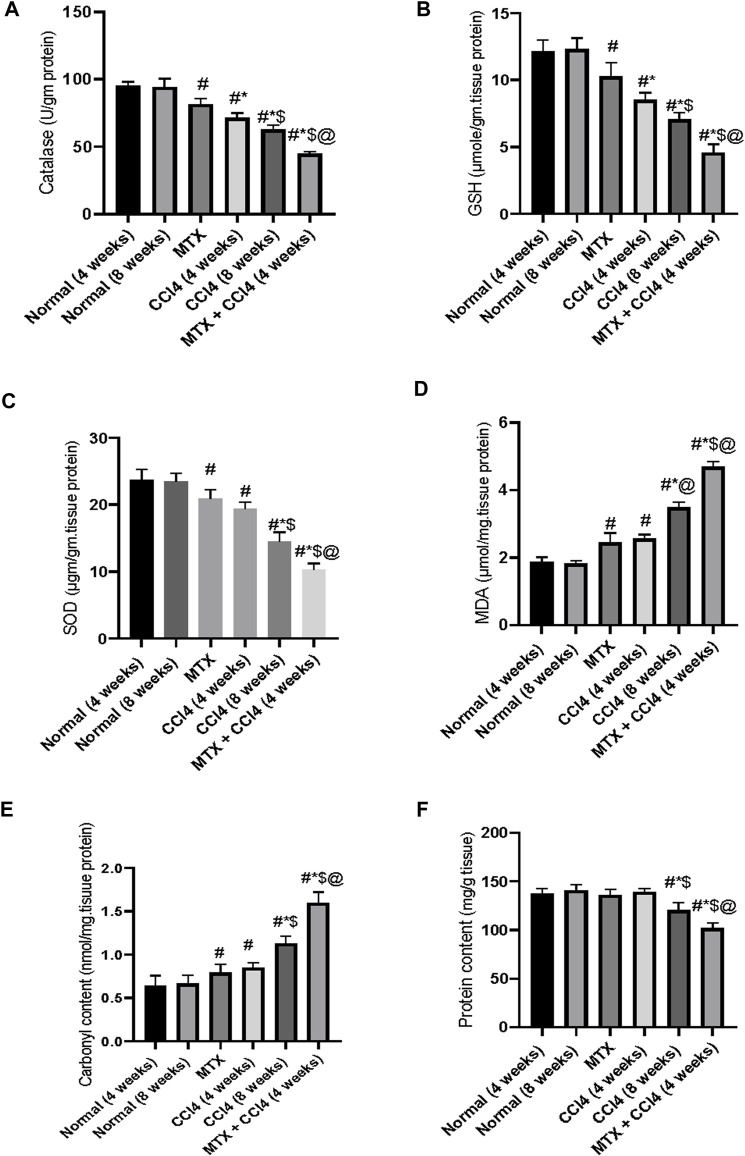
Variations in the levels of biochemical parameters in the normal and treated rats. **(A)** Analysis of catalase; **(B)** analysis of GSH; **(C)** analysis of SOD; **(D)** analysis of MDA; **(E)** analysis of carbonyl content; and **(F)** analysis of protein content. # = significantly different compared to the normal group, * = significantly different compared to the MTX group, $ = significantly different compared to the CCl4 (4 weeks) group, and @ = significantly different compared to the CCl4 (8 weeks) group. Results are presented as mean ± SEM (*n* = 14).

As shown in Figure 8B, the i.p. administration of MTX moderately decreased the hepatic tissue content of GSH (10.36 ± 0.87 μmol/g protein) compared to the normal non-treated group (12.16 ± 1.02 μmol/g protein). On the other hand, the i.p. administration of CCl_4_ (4 weeks) significantly (*p* < 0.05) decreased GSH compared to the normal group. The most prominent reduction of GSH hepatic content was observed in the CCl_4_ (8 weeks) (7.06 ± 0.78 μmol/g protein) and MTX + CCl_4_ (4.58 ± 0.37 μmol/g protein) groups. The hepatic tissue total protein content was determined as shown in Figure 8F.

### 3.8 Effect on the hepatic antioxidant enzymes

The i.p. administration of MTX resulted in a significant (*p* < 0.05) decrease in the hepatic antioxidant enzymes SOD (20.75 ± 1.9 µgm/gm protein) and catalase (81.73 ± 6.7 U/gm tissue) Figures 8A, C. The CCl_4_ (4 weeks) group showed a further reduction of both enzymes, with SOD levels of 19.4 ± 1.44 µgm/gm protein and catalase levels of 71.85 ± 6.2 U/gm tissue, compared to both the normal and MTX groups. The most severe reduction in both SOD (10.75 ± 0.72 µgm/gm protein) and catalase (44.26 ± 3.1 U/gm tissue) enzymes was observed when MTX was concomitantly administered with CCl_4_, as shown in Figures 8A, C.

### 3.9 Effect on hepatic tissue damage parameters

The i.p. administration of MTX significantly (*p* < 0.05) increased the hepatic tissue concentrations of both MDA (2.44 ± 0.16 μmol/mg of protein) and carbonyl content (0.79 ± 0.02 nmol/mg of protein) compared to the normal group. These levels were significantly different from the CCl_4_ (4 weeks) group. On the other hand, the i.p. administration of CCl_4_ (8 weeks) significantly (*p* < 0.05) increased MDA (3.84 ± 0.25 μmol/mg of protein) and carbonyl content (1.12 ± 0.09 nmol/mg of protein) compared to the CCl_4_ (4 weeks) and MTX groups. The highest levels of both MDA (4.6 ± 0.31 μmol/mg of protein) and tissue carbonyl content (1.6 ± 0.14 nmol/mg of protein) were observed at the MTX + CCl_4_ group, which was significantly (*p* < 0.05) higher than other treated groups, as shown in Figures 8D, E.

### 3.10 Effect on hepatic enzyme activities

**FIGURE 9:**
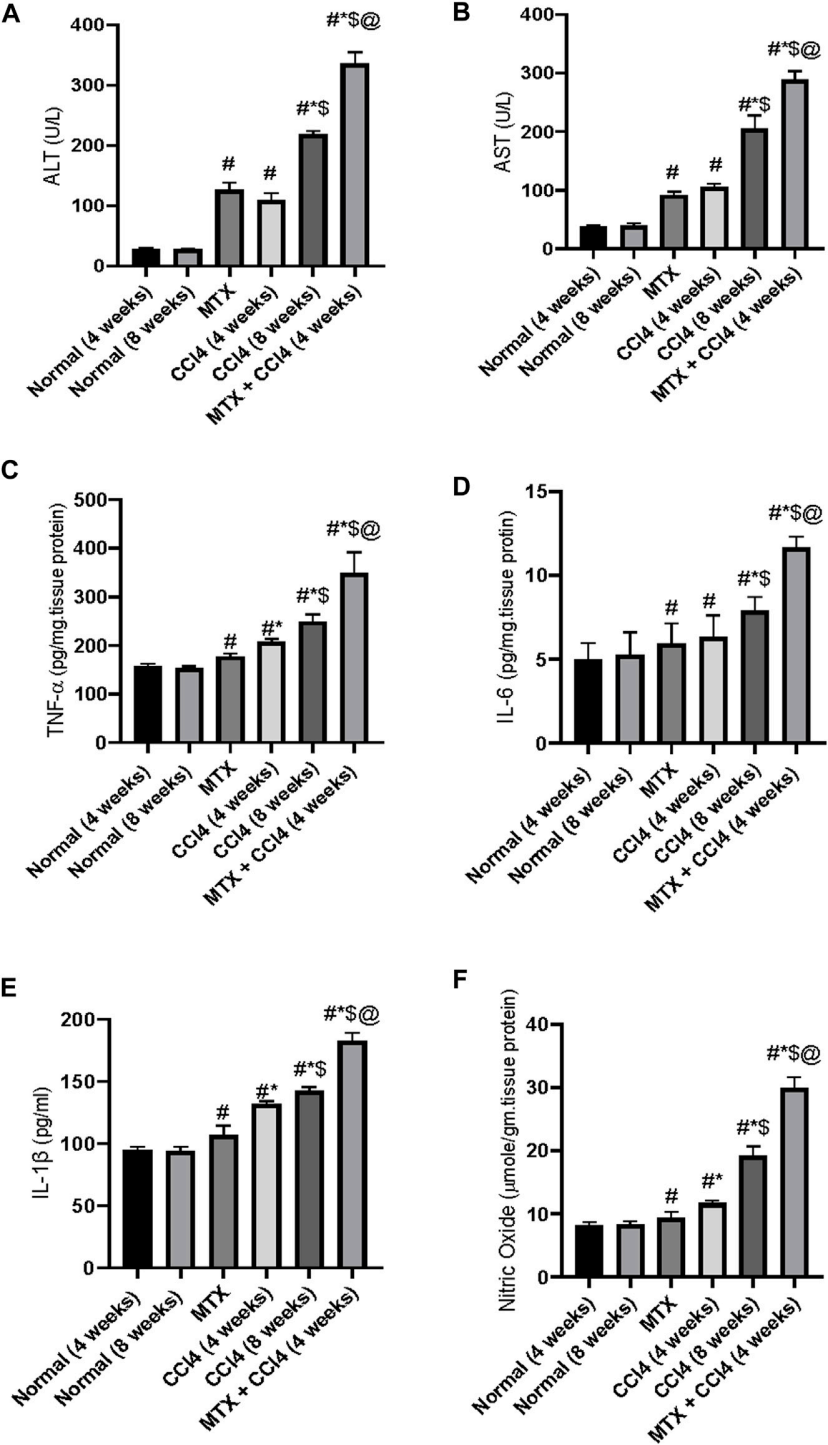
Variations in the levels of hepatic liver enzymes **(A, B)** and inflammatory cytokines **(C–F)** in the normal and treated rats. # = significantly different compared to the normal group, * = significantly different compared to the MTX group, $ = significantly different compared to the CCl_4_ (4 weeks) group, and @ = significantly different compared to the CCl_4_ (8 weeks) group. Results are presented as mean ± SEM (*n* = 14).

Both hepatic enzymes activities of AST and ALT were significantly (*p* < 0.05) increased by CCl_4_ administration for 4 weeks (108.3 ± 9.2 U/L and 121 ± 9 U/L, respectively) compared to the normal group (28.6 ± 2.4 U/L and 37.5 ± 2.2 U/L, respectively). Animals treated with CCl_4_ for 8 weeks showed higher concentrations (218.5 ± 19.2 U/L and 204.41 ± 15.2 U/L, respectively). The i.p. administration of MTX produced a significant (*p* < 0.05) elevation of ALT and AST up to 126.5 ± 9.1 U/L and 91.5 ± 7.9 U/L, respectively. A synergistic and intense up-regulatory effect on ALT and AST was observed by the combined administration of MTX with CCl_4_ for 4 weeks (335.6 ± 9.1 U/L and 288.08 ± 20.2 U/L, respectively), as shown in Figures 9A, B.

### 3.11 Effect on nitric oxide

The levels of NO were significantly (*p* < 0.05) increased by the administration of MTX (9.41 ± 0.62 μmole/gm of tissue) and CCl_4_ (11.6 ± 0.8 μmole/gm of tissue for 4 weeks and 19.2 μmole/gm of tissue for 8 weeks) compared to the normal non-treated group (8.1 μmole/gm of tissue). Additionally, the concomitant administration of MTX with CCl_4_ significantly (*p* < 0.05) produced an elevation of NO (29.9 ± 2.1 μmole/gm of tissue), which was much higher than the individual administration of both agents, as shown in Figure 9F.

### 3.12 Effect on the inflammatory cytokines

The i.p. administration of CCl_4_ (4 weeks) significantly (*p* < 0.05) increased the hepatic tissue content of tumor necrosis factor-alpha (TNF-α) and interleukin-6 (IL-6) and serum concentration of cytokine IL-1β (208.8 ± 17.9 pg/mg of tissue protein, 6.33 ± 0.5 pg/mg of tissue protein, and 131.6 ± 11.2 pg/mL, respectively) compared to the normal group (157.8 ± 12.2 pg/mg of tissue protein, 5.0 ± 0.3 pg/mg of tissue protein, and 94.7 ± 6.6 pg/mL, respectively). A synergistic proinflammatory effect was observed by the combined administration of both MTX and CCl_4_, as evidenced by an intense increase in the inflammatory cytokines TNF-α, IL-6, and IL-1β. Their levels were increased up to 349 ± 21 pg/mg of tissue protein, 11.66 ± 0.93 pg/mg of tissue protein, and 182.9 ± 12.2 pg/mL, respectively, which were significantly (*p* < 0.05) higher than those of the CCl_4_ (8 weeks) group, as shown in Figures 9C–E.

### 3.13 Mortalities between treatment groups

**FIGURE 10:**
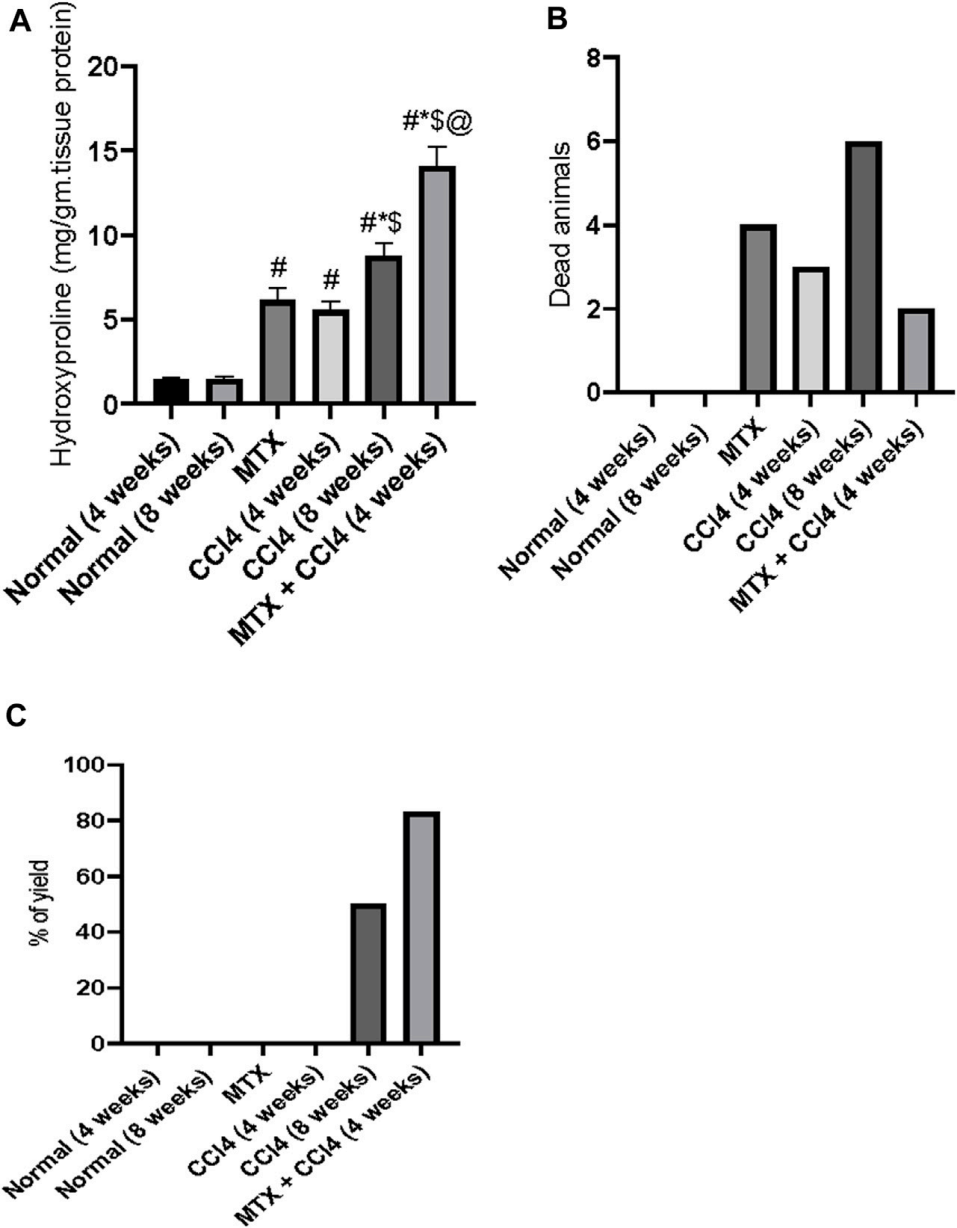
Effect of methotrexate, carbon tetrachloride, and their combined administration on **(A)** hydroxyproline, **(B)** animal mortalities, and **(C)** percent of animals with cirrhotic changes. # = significantly different compared to the normal group, * = significantly different compared to the MTX group, $ = significantly different compared to the CCl_4_ (4 weeks) group, and @ = significantly different compared to the CCl4 (8 weeks) group. Results are presented as mean ± SEM (*n* = 14).

As shown in Figure 10B, the mortality rates varied between the differently treated animal groups. There were no mortalities in the normal groups (4 and 8 weeks). The MTX group had a mortality rate of about 29%. The i.p. administration of CCl_4_ for 4 weeks caused a mortality rate of about 21%, which increased to 43% in the CCl_4_ (8 weeks) group. However, when the previously administered doses of both MTX and CCl_4_ were reduced by 50% and simultaneously injected, the mortality rates were reduced to about 14% of those of the treated animals.

### 3.14 The yield of animals with cirrhotic liver

In this study, the i.p. administration of MTX or CCl_4_ (4 weeks) did not cause any cirrhotic liver changes. However, the i.p. administration of CCl_4_ for 8 weeks induced cirrhotic liver changes in about 43% of treated animals. The combined administration of MTX with CCl_4_ (4 weeks) remarkably increased the percentage of cirrhotic animals to more than 80%, as shown in Figure 10C.

### 3.15 Effect of different treatments on hepatic content of hydroxyproline

The hydroxyproline concentration in the hepatic tissue of normal groups was found to be about 1.44 mg/g of protein. The i.p. administration of MTX and CCl_4_ (4 weeks) significantly (*p* < 0.05) increased the hydroxyproline concentration to 6.15 mg/g of protein and 5.55 mg/g of protein, respectively. The i.p. administration of CCl_4_ for 8 weeks also significantly increased the hydroxyproline concentration, compared to the normal group, up to 8.79 mg/g of protein. However, this concentration was significantly (*p* < 0.05) lower than the concentrations found in the MTX + CCl_4_ (4 weeks) group, as shown in Figure 10A.

## 4 Discussion

Liver cirrhosis is a major health problem and one of the top 10 causes of death worldwide. It is a predisposing factor for hepatic failure and HCC (Nartey et al., 2022). The underlying etiologies are various, such as the administration of some drugs, viral infection, alcohol consumption, fatty liver, and some autoimmune diseases. Hence, the underlying mechanisms vary according to the causative agent (Ye et al., 2022). Therefore, finding a proper animal model of liver cirrhosis is essential for the in-depth study of pathogenesis and a necessary means for scientists to develop therapies to prevent and treat the disease.

Ideally, animal models should mimic all features of human liver cirrhosis, including the underlying causes. However, there is currently no single model that can justify all these criteria (Tropskaya et al., 2020; Chen et al., 2021).

The CCl_4_ model is one of the most widely used chemically induced cirrhotic animal models. It is standardized and easy to conduct, but it has several drawbacks, including a long induction time (at least 8 weeks), a high mortality rate, and a low yield of animals that develop cirrhotic liver changes (Tropskaya et al., 2020). Additionally, the CCl_4_ model induces hepatocyte necrosis rather than apoptosis, which is commonly found in human patients.

Other models, such as high-fat diets, show more similarity to the human pathology, but they take much longer than the CCl_4_ model to induce cirrhotic changes and require specific transgenic animals. Alcoholic induction of hepatic cirrhosis has been challenging to induce in rodents due to minimal hepatic stellate cell activation and fibrosis (Tropskaya et al., 2020; Chen et al., 2021).

Therefore, the present study aimed to introduce a new cirrhotic animal model that can yield a higher number of animals with cirrhotic liver, be less time-consuming, and have a lower number of mortalities. The presented model anticipated a synergistic hepatic damaging effect that can lead to liver cirrhosis by the combined administration of CCl_4_ and MTX.

The intraperitoneal (i.p.) administration of CCl_4_ induces hepatic centrilobular necrosis by its reactive free radical metabolites, which are generated by CCl_4_ metabolism via cytochrome P-450 2E1 in centrilobular hepatocytes. This leads to the activation of hepatic stellate cells (HSCs) as a wound healing response, followed by hepatic tissue repair (Uehara et al., 2021). However, repeated administration of CCl_4_ can induce continuous activation of HSCs, leading to fibrosis and cirrhosis if CCl_4_ is given for more than 8 weeks (Shi et al., 2013).

In the present study, i.p. administration of CCl_4_ for 4 weeks induced mild hepatic fibrotic changes. These changes were more noticeable with continuous administration of CCl_4_ for up to 8 weeks, which resulted in mild cirrhotic liver changes, as shown by histopathological examination of H&E and Masson’s trichrome stain.

On the other hand, the combined administration of MTX with CCl_4_ for 4 weeks showed a more prominent cirrhotic liver change characterized by vascularized fibrotic septa that linked different portal tracts with each other and with hepatic central veins. This produced hepatocyte islands surrounded by fibrotic septa devoid of a central vein in addition to apoptotic liver changes, which mimicked human liver cirrhosis.

One of the biggest challenges of the CCl_4_ model for induction of experimental liver cirrhosis is the high mortality rate in animals, increasing cost, and animal suffering (Regimbeau et al., 2008; Tropskaya et al., 2020). In the present study, the mortality rate in the CCl_4_ (8 weeks) group was found to be about 43%. To overcome this challenge, we used half doses of CCl_4_ and MTX and administered them in combination. We found a significant reduction in mortality, which was about 14%.

Using CCl_4_ for 8 weeks as a model to induce liver cirrhosis always has a low to moderate yield of animals with cirrhotic liver (Fortea et al., 2018). This was consistent with our findings, as we found that only about 50% of animals in the CCl_4_ (8 weeks) group had cirrhotic liver changes. On the other hand, the combined administration of CCl_4_ with MTX yielded about 83% of animals with cirrhotic liver.

CCl_4_ is metabolized in the liver to a highly reactive chemical called the trichloromethyl radical. This radical can damage cells by reacting with nucleic acids, proteins, and lipids. This damage can lead to a variety of problems, including lipid peroxidation, decreased membrane permeability, and generalized hepatic damage characterized by fibrotic and cirrhotic liver changes (Dong et al., 2016).

In the present study, we found that intraperitoneal (i.p.) administration of CCl_4_ for 8 weeks significantly increased the hepatic tissue content of both MDA and carbonyl content, which are metabolites of lipid peroxidation and protein peroxidation, respectively. These changes are crucial features of liver fibrotic and cirrhotic changes. Furthermore, we found a substantial increase in the hepatic tissue concentrations of MDA and carbonyl protein when MTX was co-administered with CCl_4_, which suggests that MTX and CCl_4_ have a synergistic effect on liver fibrosis and cirrhosis.

Accumulating evidence suggests that inflammatory cytokines play a major role in the progression of liver cirrhosis (Dirchwolf and Ruf, 2015). Studies have proposed that tumor necrosis factor-alpha (TNF-α), produced by Kupffer cells and macrophages, plays a key role in the pathogenesis of liver cirrhosis. TNF-α can accelerate liver progenitor cell (LPC) transformation and expansion by inducing chromosomal instability in LPCs and promoting their self-renewal, which drives the conversion of LPCs into cirrhotic and cancerous cells (Yang and Seki, 2015). Furthermore, TNF-α can act as a potent activator of the proinflammatory and proapoptotic pathways (Yang and Seki, 2015). In the present study, the combined administration of MTX with CCl_4_ for 4 weeks induced an intense upregulation in the hepatic tissue content of TNF-α compared to the CCl_4_ (8 weeks) group.

During hepatic injury, macrophages and infiltrating blood cells produce the following cytokines: IL-1β, IL-6, and NF-κB-p65. These cytokines increase the expression of α-SMA, collagen I, and collagen III in both human and animal models. This indicates that these cytokines play a role in the activation of HSCs, which can lead to fibrotic and cirrhotic liver changes (Kong et al., 2012; Tian et al., 2021).

Activated inflammatory cells also stimulate the production of fibrogenic cytokines and growth factors, which further activate HSCs. This plays an important role in the development of hepatic cirrhosis (Koyama and Brenner, 2017).

Finally, it has been found that the serum or hepatic tissue levels of IL-1B, IL-6, and NF-κB-p65 are directly proportional to the cirrhotic score in animal models. This suggests that these cytokines may be used as biomarkers for the diagnosis and monitoring of hepatic cirrhosis (Koyama and Brenner, 2017).

Using immunostaining and ELISA techniques, we found that both MTX and CCl_4_ (4 weeks) intraperitoneal (i.p.) administration produced a marginal increase in the concentrations of TNF-α, IL-1β, IL-6, and NF-κB p65 inflammatory cytokines. However, when MTX was co-administered with CCl_4_, there was a remarkable synergism that caused an intense elevation of these cytokines, which were significantly higher than in the CCl_4_ (8 weeks) group. This suggests that the combination of MTX and CCl_4_ has a pro-cirrhotic effect.

ROS are highly reactive molecules that can readily interact with all cellular components of hepatocytes. ROS can cleave the phosphodiester bonds in cellular RNA and DNA and induce lipid peroxidation, which disrupts the membrane structure of hepatocytes and leads to necrosis and programmed cell death (apoptosis) (Luangmonkong et al., 2018; Abrahamovych et al., 2020).

In addition, ROS production contributes to fibrogenesis by increasing the production of cytokines, such as interleukin-6 (IL-6) and tumor necrosis factor-alpha (TNF-α), which leads to hepatic injury (Luangmonkong et al., 2018). This was consistent with our findings.

In the present study, we found that coadministration of CCl_4_ and MTX significantly decreased the activity of the antioxidant enzymes SOD and catalase, depleted the tissue content of GSH, and increased the production of the pro-oxidant nitric oxide. This led to the development of intense hepatic oxidative stress.

Previous studies have shown that CCl_4_ can induce hepatocellular cirrhosis by inducing the production of ROS and depleting antioxidant enzymes (Abdelghffar et al., 2022). MTX can also induce hepatotoxicity by generating ROS and nitrogen species, which can inhibit cytosolic NADP-dependent dehydrogenase and NADP malic enzyme, depleting GSH, SOD, and catalase hepatic reserves (Roghani et al., 2020). These findings are consistent with our results.

Bcl-2 family proteins are a major regulator of cellular apoptosis and are identified as anti-apoptotic proteins. Bcl-2 can protect hepatocytes from death induced by different insults, such as ROS and xenobiotics (Song et al., 2020). Furthermore, Bcl-2 depletion was found to play a significant role in the development of liver fibrosis, as activated HSCs are resistant to most proapoptotic stimuli due to high Bcl-2 expression. This was consistent with our findings, where we found nearly an absence of the hepatic tissue content of Bcl-2 in the MTX + CCl_4_ group, while a significant protein expression of Bcl-2 protein was observed in the normal group, and the CCl_4_ (8 weeks) group showed weak Bcl-2 protein expression (Song et al., 2020).

Quantitative biochemical methods are essential for determining the degree of fibrosis and cirrhosis in hepatic tissue when more precise data are required (Cioarca-Nedelcu et al., 2021). Hepatic cirrhosis develops mainly from the transformation of proline to procollagen by posttranslational hydroxylation to hydroxyproline. Therefore, any change in the hepatic tissue content of these amino acids is correlated with the rates of collagen formation and can be used in the assessment of the degree of liver cirrhosis (Gabr et al., 2017).

In the present study, the hepatic tissue content of hydroxyproline was dramatically increased by the combined administration of MTX with CCl_4_ for 4 weeks, which was significantly higher than the other treated groups. These results were consistent with our histopathological observations using Masson’s trichrome stain.

The present study established a new validated HPLC method for the determination of methotrexate (MTX) in hepatic tissues to examine the hypothesis that MTX can induce liver fibrosis and cirrhosis. The developed method used a micellar mobile phase to enable direct injection of hepatic homogenate samples onto the HPLC system without prior protein precipitation. The developed method is distinguished by its use of small sample volumes, lack of extraction procedure, use of micellar mobile phase, and direct injection of samples onto the chromatograph. The ability to measure MTX in small liver homogenate volumes will support the findings of the study.

In this regard, CCl_4_ could augment the MTX effect on the liver by increasing its hepatic tissue content via decreasing its hepatic excretion. The study found that MTX hepatic storage was significantly increased by combined administration with CCl4 compared to the CCl_4_ non-treated group. This suggests that MTX cirrhotic effect was dramatically increased by CCl_4_.

## 5 Conclusion

**FIGURE 11:**
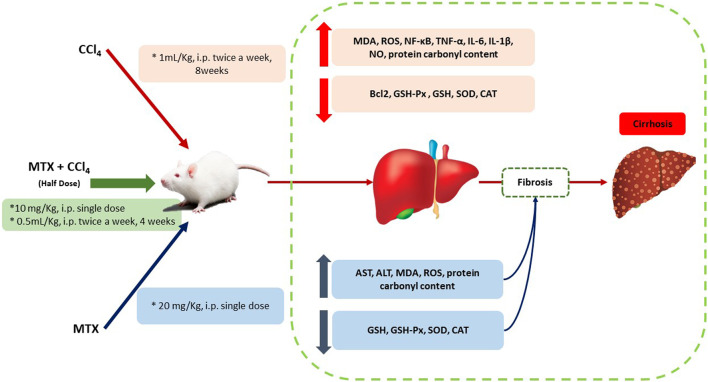
Schematic diagram of the proposed mechanisms by which CCl_4_ +MTX (half dose compared to other models) induced liver injury, inflammation, fibrosis, and cirrhosis.

The present study introduced a new experimental animal model of liver cirrhosis that overcomes some major drawbacks of previously developed models. In the presented model, the mortality rate and time for induction were remarkably decreased, along with a significant increase in the number of animals with cirrhotic liver (yield) compared to other chemically induced methods (Figure 11). In addition, new pathological features that mimic human cirrhosis were determined, which were not found in previously described chemical methods. The present model can save time, cost, and animal suffering compared to other chemically induced methods.

## Data Availability

The original contributions presented in the study are included in the article/Supplementary Material; further inquiries can be directed to the corresponding author.

## Abbreviations

ALT, alanine aminotransferase; AST, aspartate aminotransferase; CAT, catalase activity; CCl_4,_ carbon tetrachloride; GSH, glutathione; H&E, hematoxylin and eosin; HETP, height equivalent to a theoretical plate; ICH, International Council for Harmonization; IL-1β, interleukin-1β; IL-6, interleukin-6; LOD, limit of detection; LOQ, limit of quantitation; MDA, malondialdehyde; MLC, micellar liquid chromatography; MTX, methotrexate; NADPH, reduced nicotinamide adenosine diphosphate hydrogen; NF-κB, nuclear factor kappa B; NO, nitrous oxide; ROS, reactive oxygen species; SDS, sodium dodecyl sulfate; SOD, superoxide dismutase; TAA, thioacetamide; TNF-α, tumor necrosis factor-α; α-SMA, α-smooth muscle actin.

## References

[B1] Abdelghffar E. A. Obaid W. A. Alamoudi M. O. Mohammedsaleh Z. M. Annaz H. Abdelfattah M. A. O. (2022). Thymus fontanesii attenuates CCl4-induced oxidative stress and inflammation in mild liver fibrosis. Biomed. Pharmacother. 148, 112738. 10.1016/j.biopha.2022.112738 35202909

[B2] Aboubakr E. M. Mohammed H. A. Hassan A. S. Mohamed H. B. Dosoky M. I. E. Ahmad A. M. (2022). Glutathione-loaded non-ionic surfactant niosomes: A new approach to improve oral bioavailability and hepatoprotective efficacy of glutathione. Nanotechnol. Rev. 11, 117–137. 10.1515/ntrev-2022-0010

[B3] Abrahamovych M. Abrahamovych O. Fayura O. Fayura L. Tolopko S. (2020). The effect of oxidative stress on the autonomic nervous system in patients with liver cirrhosis. Georgian Med. News 99, 94. 32141858

[B4] Ali F. E. M. Bakr A. G. Abo-Youssef A. M. Azouz A. A. Hemeida R. A. M. (2018). Targeting Keap-1/Nrf-2 pathway and cytoglobin as a potential protective mechanism of diosmin and pentoxifylline against cholestatic liver cirrhosis. Life Sci. 207, 50–60. 10.1016/j.lfs.2018.05.048 29852187

[B5] Aslan A. Gok O. Erman O. Kuloglu T. (2018). Ellagic acid impedes carbontetrachloride-induced liver damage in rats through suppression of NF-kB, Bcl-2 and regulating Nrf-2 and caspase pathway. Biomed. Pharmacother. 105, 662–669. 10.1016/j.biopha.2018.06.020 29902765

[B6] Bae S. H. Choi H. G. Park S. Y. Chang S. Y. Kim H. Kim S. H. (2022). Effects of isosakuranetin on pharmacokinetic changes of tofacitinib in rats with N-Dimethylnitrosamine-Induced liver cirrhosis. Pharmaceutics 14, 2684. 10.3390/pharmaceutics14122684 36559177 PMC9783783

[B7] Bhavna, Ojha A. Bhargava S. (2022). “Chapter 3 - international Council for harmonisation (ICH) guidelines,” in Regulatory affairs in the pharmaceutical industry ALI, J. BABOOTA S. (Germany: Academic Press).

[B8] Bode J. G. Albrecht U. Haussinger D. Heinrich P. C. Schaper F. (2012). Hepatic acute phase proteins--regulation by IL-6- and IL-1-type cytokines involving STAT3 and its crosstalk with NF-κB-dependent signaling. Eur. J. Cell Biol. 91, 496–505. 10.1016/j.ejcb.2011.09.008 22093287

[B9] Bryan N. S. Grisham M. B. (2007). Methods to detect nitric oxide and its metabolites in biological samples. Free Radic. Biol. Med. 43, 645–657. 10.1016/j.freeradbiomed.2007.04.026 17664129 PMC2041919

[B10] Chatamra K. Proctor E. (1981). Phenobarbitone-induced enlargement of the liver in the rat: Its relationship to carbon tetrachloride-induced cirrhosis. Br. J. Exp. Pathol. 62, 283–288. 7248170 PMC2041698

[B11] Chen Z. Li S. Han L. He X. (2021). Optimized protocol for an inducible rat model of liver tumor with chronic hepatocellular injury, inflammation, fibrosis, and cirrhosis. Star. Protoc. 2, 100353. 10.1016/j.xpro.2021.100353 33665633 PMC7905469

[B12] Cioarca-Nedelcu R. Atanasiu V. Stoian I. (2021). Alcoholic liver disease-from steatosis to cirrhosis - a biochemistry approach. J. Med. Life 14, 594–599. 10.25122/jml-2021-0081 35027961 PMC8742892

[B13] Das K. K. Gupta A. D. Dhundasi S. A. Patil A. M. Das S. N. Ambekar J. G. (2006). Effect of L-ascorbic acid on nickel-induced alterations in serum lipid profiles and liver histopathology in rats. J. Basic Clin. Physiol. Pharmacol. 17, 29–44. 10.1515/jbcpp.2006.17.1.29 16639878

[B14] Desmyter L. Fan Y. D. Praet M. Jaworski T. Vervecken W. De Hemptinne B. (2007). Rating of CCl(4)-induced rat liver fibrosis by blood serum glycomics. J. Gastroenterol. Hepatol. 22, 1148–1154. 10.1111/j.1440-1746.2006.04553.x 17608861

[B15] Dirchwolf M. Ruf A. E. (2015). Role of systemic inflammation in cirrhosis: From pathogenesis to prognosis. World J. Hepatol. 7, 1974–1981. 10.4254/wjh.v7.i16.1974 26261687 PMC4528271

[B16] Dong S. Chen Q. L. Song Y. N. Sun Y. Wei B. Li X. Y. (2016). Mechanisms of CCl4-induced liver fibrosis with combined transcriptomic and proteomic analysis. J. Toxicol. Sci. 41, 561–572. 10.2131/jts.41.561 27452039

[B17] El-Kashef D. H. Serrya M. S. (2019). Sitagliptin ameliorates thioacetamide-induced acute liver injury via modulating TLR4/NF-KB signaling pathway in mice. Life Sci. 228, 266–273. 10.1016/j.lfs.2019.05.019 31077717

[B18] Fortea J. I. Fernandez-Mena C. Puerto M. Ripoll C. Almagro J. Banares J. (2018). Comparison of two protocols of carbon tetrachloride-induced cirrhosis in rats - improving yield and reproducibility. Sci. Rep. 8, 9163. 10.1038/s41598-018-27427-9 29907790 PMC6003930

[B19] Gabr S. A. Alghadir A. H. Sherif Y. E. Ghfar A. A. (2017). “Hydroxyproline as a biomarker in liver disease,” in Biomarkers in liver disease PATEL, V. B. PREEDY V. R. (Dordrecht: Springer Netherlands).

[B20] Garcia D. S. Saturansky E. I. Poncino D. Martinez-Artola Y. Rosenberg S. Abritta G. (2019). Hepatic toxicity by methotrexate with weekly single doses associated with folic acid in rheumatoid and psoriatic arthritis. What is its real frequency? Ann. Hepatol. 18, 765–769. 10.1016/j.aohep.2019.01.011 31105018

[B21] Gieling R. G. Wallace K. Han Y. P. (2009). Interleukin-1 participates in the progression from liver injury to fibrosis. Am. J. Physiol. Gastrointest. Liver Physiol. 296, G1324–G1331. 10.1152/ajpgi.90564.2008 19342509 PMC2697947

[B22] Huang X.-J. Choi Y.-K. Im H.-S. Yarimaga O. Yoon E. Kim H.-S. (2006). Aspartate aminotransferase (AST/GOT) and alanine aminotransferase (ALT/GPT) detection techniques. Sensors 6, 756–782. 10.3390/s6070756

[B23] Kleiner D. E. Brunt E. M. Van Natta M. Behling C. Contos M. J. Cummings O. W. (2005). Design and validation of a histological scoring system for nonalcoholic fatty liver disease. Hepatology 41, 1313–1321. 10.1002/hep.20701 15915461

[B24] Knockaert L. Berson A. Ribault C. Prost P.-E. Fautrel A. Pajaud J. (2012). Carbon tetrachloride-mediated lipid peroxidation induces early mitochondrial alterations in mouse liver. Lab. Investig. 92, 396–410. 10.1038/labinvest.2011.193 22157718

[B25] Kong X. Horiguchi N. Mori M. Gao B. (2012). Cytokines and STATs in liver fibrosis. Front. Physiol. 3, 69. 10.3389/fphys.2012.00069 22493582 PMC3318231

[B26] Koyama Y. Brenner D. A. (2017). Liver inflammation and fibrosis. J. Clin. Invest. 127, 55–64. 10.1172/JCI88881 28045404 PMC5199698

[B27] Kruger N. J. (1994). The Bradford method for protein quantitation. Methods Mol. Biol. 32, 9–15. 10.1385/0-89603-268-X:9 7951753

[B28] Lai Y. H. Chen L. J. Cheng J. T. (2013). Role of TNF-alpha in renal damage in mice showing hepatic steatosis induced by high fat diet. Horm. Metab. Res. 45, 38–42. 10.1055/s-0032-1321871 22956307

[B29] Lin Z. Cai F. Lin N. Ye J. Zheng Q. Ding G. (2014). Effects of glutamine on oxidative stress and nuclear factor-κB expression in the livers of rats with nonalcoholic fatty liver disease. Exp. Ther. Med. 7, 365–370. 10.3892/etm.2013.1434 24396406 PMC3881322

[B30] Luangmonkong T. Suriguga S. Mutsaers H. A. M. Groothuis G. M. M. Olinga P. Boersema M. (2018). “Targeting oxidative stress for the treatment of liver fibrosis,” in Reviews of physiology, biochemistry and Pharmacology NILIUS B. DE TOMBE P. GUDERMANN T. JAHN R. LILL R. (Cham: Springer International Publishing), 175. 10.1007/112_2018_10 29728869

[B31] Missaoui N. Landolsi H. Mestiri S. Essakly A. Abdessayed N. Hmissa S. (2019). Immunohistochemical analysis of c-erbB-2, Bcl-2, p53, p21WAF1/Cip1, p63 and Ki-67 expression in hydatidiform moles. Pathology - Res. Pract. 215, 446–452. 10.1016/j.prp.2018.12.015 30558966

[B32] Nartey Y. A. Antwi S. O. Bockarie A. S. Hiebert L. Njuguna H. Ward J. W. (2022). Mortality burden due to liver cirrhosis and hepatocellular carcinoma in Ghana; prevalence of risk factors and predictors of poor in-hospital survival. PLoS One 17, e0274544. 10.1371/journal.pone.0274544 36099308 PMC9469955

[B33] Natarajan S. K. Thomas S. Ramamoorthy P. Basivireddy J. Pulimood A. B. Ramachandran A. (2006). Oxidative stress in the development of liver cirrhosis: A comparison of two different experimental models. J. Gastroenterol. Hepatol. 21, 947–957. 10.1111/j.1440-1746.2006.04231.x 16724977

[B55] Nickovic V. P. Miric D. Kisic B. Kocic H. Stojanovic M. Buttice S. (20181). Oxidative stress, NOx/l-arginine ratio and glutathione/glutathione S-transferase ratio as predictors of ‘sterile inflammation’ in patients with alcoholic cirrhosis and hepatorenal syndrome type II. Ren Fail 40 (1), 340–349. 10.1080/0886022X.2018.1459699 29658815 PMC6014490

[B34] Noeman S. A. Hamooda H. E. Baalash A. A. (2011). Biochemical study of oxidative stress markers in the liver, kidney and heart of high fat diet induced obesity in rats. Diabetology Metabolic Syndrome 3, 17. 10.1186/1758-5996-3-17 21812977 PMC3174870

[B35] Nusrat S. Khan M. S. Fazili J. Madhoun M. F. (2014). Cirrhosis and its complications: Evidence based treatment. World J. Gastroenterol. 20, 5442–5460. 10.3748/wjg.v20.i18.5442 24833875 PMC4017060

[B36] Okado-Matsumoto A. Fridovich I. (2001). Subcellular distribution of superoxide dismutases (SOD) in rat liver: Cu,Zn-SOD in mitochondria. J. Biol. Chem. 276, 38388–38393. 10.1074/jbc.M105395200 11507097

[B37] Patyra E. Kwiatek K. (2021). Analytical capabilities of micellar liquid chromatography and application to residue and contaminant analysis: A review. J. Sep. Sci. 44, 2206–2220. 10.1002/jssc.202001261 33811781

[B56] Pomacu M. M. Trașcă M. D. Pădureanu V. Bugă A. M. Andrei A. M. Stănciulescu E. C. (2021). Interrelation of inflammation and oxidative stress in liver cirrhosis. Exp. Ther. Med. 21 (6), 602. 10.3892/etm.2021.10034 33936259 PMC8082585

[B38] Proctor E. Chatamra K. (1982). High yield micronodular cirrhosis in the rat. Gastroenterology 83, 1183–1190. 10.1016/s0016-5085(82)80126-1 7129027

[B39] Regimbeau J. M. Fuks D. Kohneh-Shahri N. Terris B. Soubrane O. (2008). Restrictive model of compensated carbon tetrachloride-induced cirrhosis in rats. World J. Gastroenterol. 14, 6943–6947. 10.3748/wjg.14.6943 19058329 PMC2773857

[B40] Roghani M. Kalantari H. Khodayar M. J. Khorsandi L. Kalantar M. Goudarzi M. (2020). Alleviation of liver dysfunction, oxidative stress and inflammation underlies the protective effect of ferulic acid in methotrexate-induced hepatotoxicity. Drug Des. Devel Ther. 14, 1933–1941. 10.2147/DDDT.S237107 PMC7250701 32546960

[B41] Runyon B. A. Sugano S. Kanel G. Mellencamp M. A. (1991). A rodent model of cirrhosis, ascites, and bacterial peritonitis. Gastroenterology 100, 489–493. 10.1016/0016-5085(91)90221-6 1985046

[B42] Sahindokuyucu-Kocasari F. Akyol Y. Ozmen O. Erdemli-Kose S. B. Garli S. (2021). Apigenin alleviates methotrexate-induced liver and kidney injury in mice. Hum. Exp. Toxicol. 40, 1721–1731. 10.1177/09603271211009964 33845614

[B43] Scholten D. Trebicka J. Liedtke C. Weiskirchen R. (2015). The carbon tetrachloride model in mice. Lab. Anim. 49, 4–11. 10.1177/0023677215571192 25835733

[B44] Shetty A. Cho W. Alazawi W. Syn W. K. (2017). Methotrexate hepatotoxicity and the impact of nonalcoholic fatty liver disease. Am. J. Med. Sci. 354, 172–181. 10.1016/j.amjms.2017.03.014 28864376

[B45] Shi H. Shi H. Kong M. Chen G. Zhao J. Ding M. (2013). Compound nutrients promote liver rehabilitation and regeneration in rats with CCl4;-induced liver cirrhosis. Xi Bao Yu Fen Zi Mian Yi Xue Za Zhi 29, 1237–1241. 24321063

[B46] Shin G.-M. Koppula S. Chae Y.-J. Kim H.-S. Lee J.-D. Kim M.-K. (2018). Anti-hepatofibrosis effect of Allium senescens in activated hepatic stellate cells and thioacetamide-induced fibrosis rat model. Pharm. Biol. 56, 632–642. 10.1080/13880209.2018.1529801 31070527 PMC6282452

[B47] Song S. Sun K. Dong J. Zhao Y. Liu F. Liu H. (2020). microRNA-29a regulates liver tumor-initiating cells expansion via Bcl-2 pathway. Exp. Cell Res. 387, 111781. 10.1016/j.yexcr.2019.111781 31857112

[B57] Tang S. Huang Z. Jiang J. Gao J. Zhao C. Tai Y. (2021). Celecoxib ameliorates liver cirrhosis via reducing inflammation and oxidative stress along spleen-liver axis in rats. Life Sci. 272, 119203. 10.1016/j.lfs.2021.119203 33577848

[B48] Tian F. Jiang T. Qi X. Zhao Z. Li B. Aibibula M. (2021). Role of cytokines on the progression of liver fibrosis in mice infected with echinococcus multilocularis. Infect. Drug Resist 14, 5651–5660. 10.2147/IDR.S344508 34992391 PMC8714463

[B49] Tropskaya N. S. Kislyakova E. A. Vilkova I. G. Kislitsyna O. S. Gurman Y. V. Popova T. S. (2020). Experimental model of cirrhosis of the liver. Bull. Exp. Biol. Med. 169, 416–420. 10.1007/s10517-020-04899-2 32748146

[B50] Uehara T. Pogribny I. P. Rusyn I. (2021). The DEN and CCl4 -induced mouse model of fibrosis and inflammation-associated hepatocellular carcinoma. Curr. Protoc. 1, 2111–e310. 10.1002/0471141755.ph1430s66 PMC8744072 34370903

[B51] Yang Y. M. Seki E. (2015). TNFα in liver fibrosis. Curr. Pathobiol. Rep. 3, 253–261. 10.1007/s40139-015-0093-z 26726307 PMC4693602

[B52] Ye F. Zhai M. Long J. Gong Y. Ren C. Zhang D. (2022). The burden of liver cirrhosis in mortality: Results from the global burden of disease study. Front. Public Health 10, 909455. 10.3389/fpubh.2022.909455 36033800 PMC9403789

[B53] Yucel Y. Oguz E. Kocarslan S. Tatli F. Gozeneli O. Seker A. (2017). The effects of lycopene on methotrexate-induced liver injury in rats. Bratisl. Lek. Listy 118, 212–216. 10.4149/BLL_2017_042 28471231

[B54] Zhou W. C. Zhang Q. B. Qiao L. (2014). Pathogenesis of liver cirrhosis. World J. Gastroenterol. 20, 7312–7324. 10.3748/wjg.v20.i23.7312 24966602 PMC4064077

